# A clinical and biomechanical comparison of INFIX plus single versus double sacroiliac screw fixation for unstable pelvic ring injury

**DOI:** 10.1186/s13018-022-03133-1

**Published:** 2022-05-21

**Authors:** Hongfen Chen, Chao Ding, Yongqiang Liu, Zhen Kong, Siling Chang, Feng Huang, Heng Li, Qingxiang Guo, Yuehua Yang, Hua Zhong, Shaozheng Yang

**Affiliations:** 1 Department of Orthopaedics, The Fifth Affiliated Hospital, Southern Medical University, No. 566 Congcheng Avenue, Conghua District, Guangzhou, 510900 People’s Republic of China; 2 Department of Orthopedics, Shanghai General Hospital, Shanghai Jiao Tong University School of Medicine, Shanghai, 200080 People’s Republic of China

**Keywords:** Pelvic fracture, INFIX, Internal fixation, Biomechanics, Sacroiliac screw fixation

## Abstract

**Background:**

The aim of this study is to compare the clinical and biomechanical outcome of INFIX plus single with two sacroiliac screw fixation for unstable pelvic fractures of Type C.

**Methods:**

Sixteen cadavers were randomly subjected to INFIX plus single or double sacroiliac screw fixations and then mounted onto the ElectroForce loading machine under different vertical loads. To investigate the clinical outcomes of the two techniques, nineteen patients were retrospectively analyzed. The main outcome measures were postoperative radiographic reduction grading (using the Tornetta and Matta grading system), functional outcome (using the Majeed scoring system), and incidence of complications.

**Results:**

In the biomechanical study, INFIX plus double sacroiliac screw fixation showed better biomechanical stability than fixation with a single sacroiliac screw (*p* < 0.05). In our clinical case series, all 19 patients had bony union 6 months after the operation. INFIX plus double sacroiliac screw fixation also demonstrated a better functional outcome and a higher radiographic satisfactory rate than INFIX plus single sacroiliac screw fixation (79.25 ± 5.47; 91.33 ± 4.97; *p* < 0.05), (77.78% vs. 60%; *p* = 0.05). One patient in INFIX plus single-screw fixation group had screw loosening at 6-month follow-up postoperatively. One case in each group suffered heterotopic ossification and the lateral femoral cutaneous nerve paralysis, and one patient suffered from infection.

**Conclusion:**

INFIX plus double sacroiliac screw fixation demonstrated more stability in cadaveric biomechanical analysis and better clinical outcomes than INFIX plus single sacroiliac screw fixation.

## Introduction

Pelvic fractures are common and associated with significant morbidity and mortality. Pelvic ring injury fracture (AO/OTA type C) is severe unstable fracture that is often accompanied by rupture of the posterior ligaments. Researchers have shown that simultaneous fixation of the anterior and posterior pelvic rings can offer adequate biomechanical stability, satisfactory healing, and functional recovery [1, 2]. By using simultaneous fixation, the complications related to single anterior or posterior pelvic ring fixation, such as pain, chronic pelvic instability, and disability, can potentially be avoided.

Traditional methods of anterior pelvic ring fixation include open reduction and internal fixation (ORIF) or external fixation. The risks of ORIF for anterior pelvic ring fractures include vascular nerve injury and inguinal hernia [3]. External fixation is mainly used in the emergency department to achieve hemodynamic stability, with potential complications including poor patient tolerance, postoperative infection, aseptic loosening, and joint dyskinesia [4–6]. Anterior subcutaneous internal fixation (INFIX) has recently been proposed by several scholars as a minimally invasive technique to treat anterior pelvic ring injury, with proposed benefits including shorter operative times, smaller wound size, less periosteal stripping, and adequate functional outcomes compared to traditional techniques [7–11].

For posterior pelvic ring injury, percutaneous sacroiliac screw fixation has emerged as a preferred technique [12]. Proposed benefits of this technique include reduced blood loss, shorter operative times, reduced tissue trauma, and fewer complications when compared to internal fixation [13, 14]. A double-screw fixation technique has been shown to provide optimal biomechanical stability compared to a single-screw fixation technique in cadaver model studies [15–17], but these findings have not been replicated in clinical studies [18]. Studies that have investigated the outcomes of combining INFIX and sacroiliac screw fixation to treat anterior and posterior pelvic ring fractures (AO/OTA type C) have shown varied and inconclusive outcomes.

The aim of our study was to compare the biomechanical stability and clinical outcomes of combining INFIX with single versus double sacroiliac screw fixation for Type C unstable pelvic fractures to determine whether a single-screw technique provides comparable outcomes to those of a double-screw technique.

## Materials and methods

### Cadaveric study

For the cadaveric research, 16 fresh human adult cadaver pelvises (8 males and 8 females; mean age ± SD: 55 ± 12.8 years) were selected for biomechanical testing at Southern Medical University and get donor families’ consent. We obtained approval from our southern medical university’s ethics committee (Number: XHEC-D-2015-112). Soft tissue was removed from each pelvis, retaining the following ligaments: anterior and posterior sacroiliac, sacrospinous, and sacrotuberous, and bones: L4, L5, sacrum, and 15 cm of each proximal femur. In addition, the specimens were scanned by X-ray to exclude pathologies such as tumors, tuberculosis, and osteoporosis. The specimens were stored in a Freezer at − 30 °C.

### Fracture model creation

**Fig. 1:**
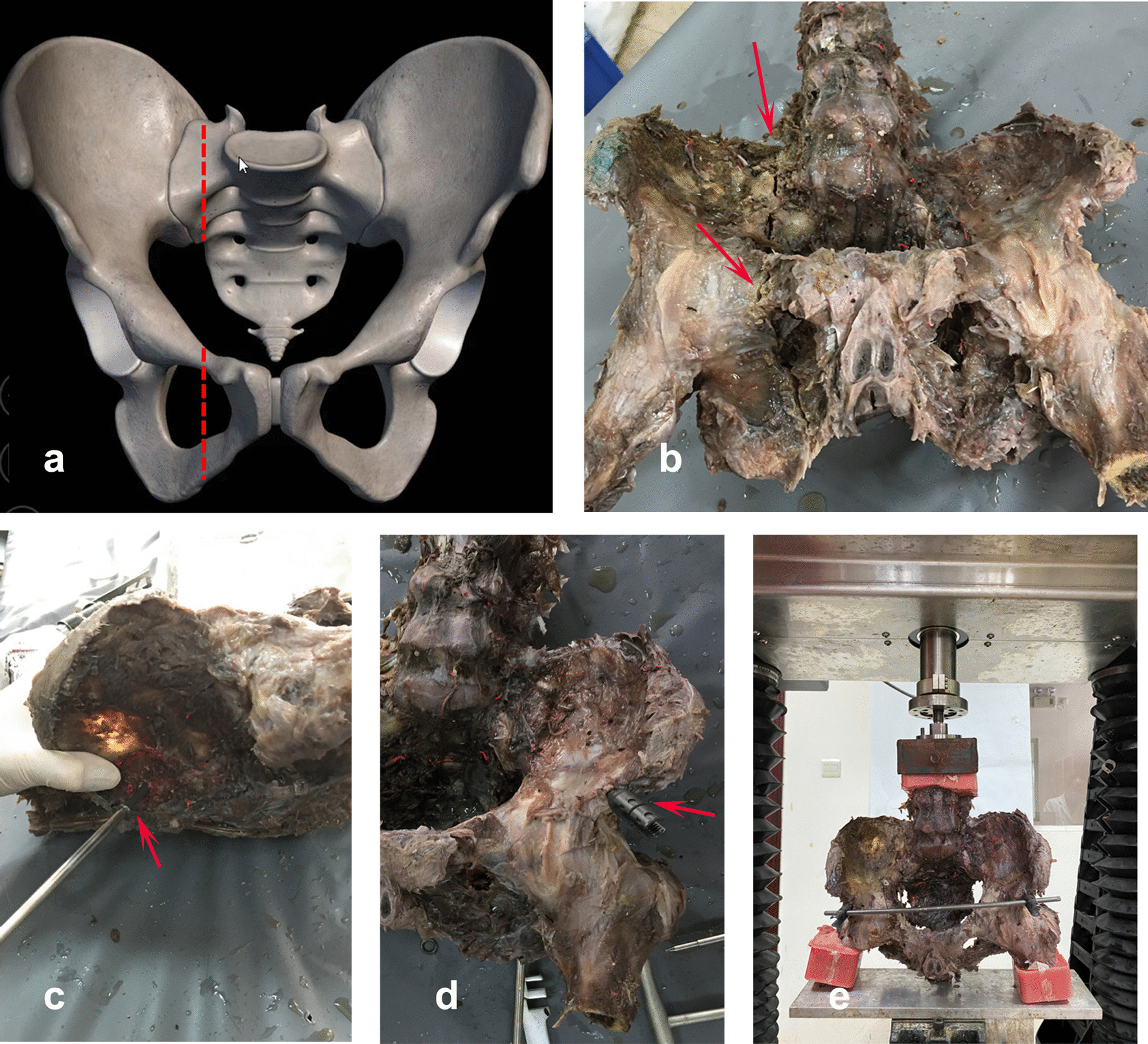
Models of AO/OTA type C1 unstable pelvic fractures were created. Pelvic fracture C1 on diagrammatic pelvic model (**a**) and fresh human adult cadaver pelvis (**b**). A sacroiliac screw was fixed at S1 (**c**), and two multiaxial pedicle screws (6.5 mm × 70 mm) were inserted through the point of each anterior inferior iliac spine (**d**). Loading of the fracture models on a biomechanical testing machine using the two-limb-stance model (**e**)

Models of AO/OTA type C1.3 unstable pelvic fractures were created by sawing each right upper and lower pubic ramus vertically with an electric pendulum saw and then sawing each ipsilateral sacrum vertically through the Denis I region (Fig. 1). The specimens were randomly divided into two treatment groups (groups A and B) of eight specimens each, with group A receiving INFIX plus single sacroiliac screw fixation at S1 and group B receiving INFIX plus double sacroiliac screw fixation at S1 and S2. All implants were placed by the same operator, and all implants were provided by Synthes. Cannulated screws were used to fix S1 (screw dimension: 7.3 mm × 105 mm) and S2 (screw dimensions: 7.3 mm × 65 mm). Two multiaxial pedicle screws (6.5 mm × 70 mm) were inserted through the point of both anterior inferior iliac spine and fixed with a titanium rod (6.0 mm × 400 mm). Then, two Kirschner wires (1.0 mm) were inserted into the vertical horizontal line at each end of the pubic ramus fracture to measure the displacement between the two wires during pelvic compression.

### Biomechanical testing

All biomechanical experiments were conducted in the Department of Anatomy in Southern Medical University’s Engineering Institute. The L5 vertebra and the distal part of each femur were embedded and immobilized within a self-congealing resin denture powder and installed in the biomechanical testing machine, ElectroForce® 3510 (Bach. Co., USA) (Fig. 1). Axial compression was applied to the base of the upper sacrum at a loading rate of 20 N/S and maintained for 30 s when the load reached 200, 400, 600, and 800 N. The distance between the two Kirschner wires on each specimen was recorded, and each loading cycle was repeated at least three times.

### Clinical study

**Fig. 2:**
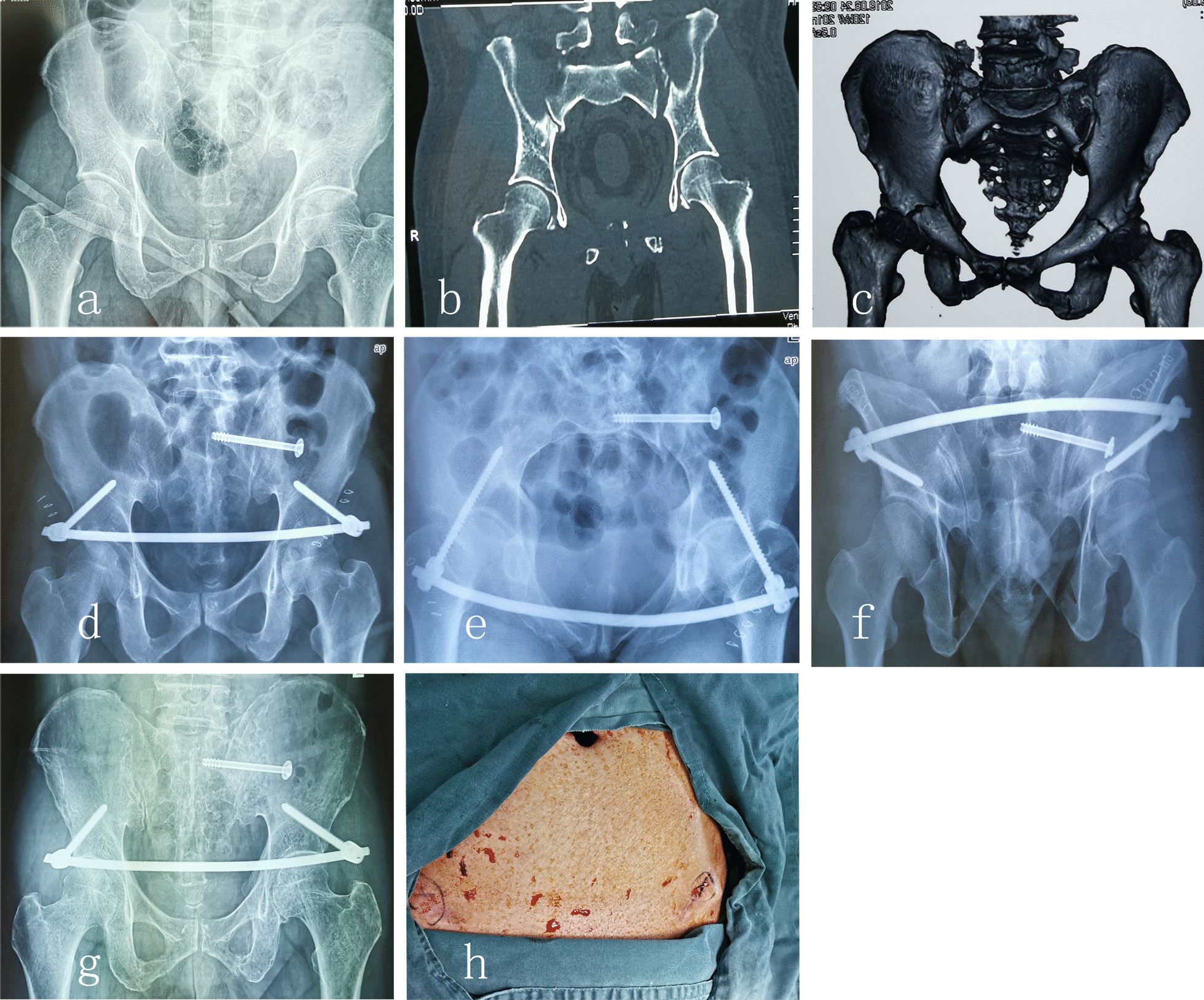
A series of images depicting the clinical course of a patient (male, 57 years, AO/OTA type C1) who developed loosening of screws at 6 months post-surgery. Preoperative images of the pelvis, including anteroposterior (**a**) and coronal (**b**) radiographs, and three-dimensional CT scan images (**c**), all depicting left sacroiliac joint fracture dislocation with complete instability, and anterior pelvis fracture with concomitant bilateral superior and inferior pubic rami fractures. Plain radiographs taken 1 day after INFIX surgery, showing anteroposterior (**d**), inlet (**e**), and outlet (**f**) views of the pelvis. Images taken 6 months after INFIX surgery, showing screw loosening on anteroposterior radiographs (**g**) and compression of the skin (**h**)

We conducted a retrospective consecutive case series of patients treated in the Department of Orthopedics at the Fifth Affiliated Hospital, Southern Medical University, from February 2018 to January 2020. Nineteen patients fulfilled inclusion criteria (a minimum follow-up period of 12 months) with OTA/AO type C pelvic ring fractures and received INFIX plus sacroiliac screw fixation during the period in review. Of these patients, group C (4 females and 6 males) received INFIX plus single sacroiliac screw fixation and group D (3 females and 6 males) received INFIX plus double sacroiliac screw fixation. Patients’ demographic data, injury severity score (ISS), injury mechanism, time to surgery, procedure time, and estimated blood loss were recorded. At each 12–24-month follow-up, the following outcome measures were recorded: postoperative radiographic reduction grading, functional outcome using the Majeed scoring system [8], and incidence of complications. Reduction quality and implant position were assessed on radiographs (anteroposterior, inlet, and outlet views of the pelvis) (Fig. 2d–f) using the Tornetta and Matta grading system, at monthly intervals for the first 6 months.

### Statistical analysis

All statistical analyses were performed using SPSS software, version 23.0 (IBM SPSS Statistics for Windows, Armonk, NY, USA). The measurement data were presented as mean ± SD and analyzed using a paired t test. The Chi-square test was used for enumeration type data, such as the incidence of complications. Statistical significance was set at *p* < 0.05.

## Results

### Cadaveric study

There was no significant difference in specimen height or weight between the two groups (*p* > 0.05). As shown in Table 1, no significant difference was found in anterior pelvic ring stability between the single- and double-screw fixation techniques when axial loading was less than 200 N. In both groups, anterior pelvis displacement increased proportionally with increased axial loading. Group B showed significantly greater stability under axial loading with 400 N (mean displacement differences: group A = 1.07 ± 0.39 mm; group B = 0.21 ± 0.09 mm; *p* < 0.001). In addition, group B showed significantly better anterior pelvis stability under axial loading with 800 N (mean displacement difference: group A = 2.37 ± 0.12 mm; group B = 1.29 ± 0.14 mm; *p* = 0.012). These findings suggest that fixation outcomes are superior when using a double-screw technique.

**Table 1:** Comparison of anterior pelvic ring and posterior pelvic ring displacement distance (mm) under 200–800 N axial loading

Parameter	A (INFIX + S1)	B (INFIX + S1S2)	*p*
Anterior pelvic ring			
200 N	0.60 ± 0.13	0.67 ± 0.04	0.201
400 N	1.07 ± 0.39	0.21 ± 0.09	< 0.001
600 N	1.63 ± 0.11	0.62 ± 0.18	< 0.001
800 N	2.37 ± 0.12	1.29 ± 0.14	< 0.001
Posterior pelvic ring			
200 N	0.30 ± 0.04	0.22 ± 0.09	0.161
400 N	0.38 ± 0.04	0.32 ± 0.07	0.187
600 N	0.46 ± 0.05	0.39 ± 0.08	0.176
800 N	0.54 ± 0.05	0.46 ± 0.05	0.096

### Clinical study

#### Baseline characteristics of patients

Nineteen patients met the inclusion criteria. Group C included 10 cases: 6 males and 4 females, mean age = 40.88 ± 11.43 years (range 28–56 years), and mean follow-up time = 20.5 months (range 12–24 months). Group D included 9 cases: 6 males and 3 females, mean age = 37.50 ± 12.36 years (range 26–50 years), and mean follow-up time = 23.2 months (range 12–27 months). The baseline characteristics of the patients are shown in Table 3. No significant inter-group differences were observed in terms of age, sex, ISS scores, fracture type, and injury mechanism (Table 2). All surgeries were performed by one group of surgeons, including a senior orthopedic pelvic trauma surgeon (Zhong Hua) and two general trauma surgeons (Chen Hongfen and Ding Chao), and a radiologist.

**Table 2:** Patient demographics of two groups (ISS, injury severity score)

Parameter	Group C (*n* = 10)	Group D (*n* = 9)	*p*
Age (years)	40.88 ± 11.43	37.50 ± 12.36	0.317
Gender: male/female	6/4	6/3	0.763
ISS	29 (20, 32)	24 (22, 29)	0.319
AO/OTA Classification			
61-C			
61-C1	4	5	
61-C2	6	4	0.497
Injury mechanism			
Fall from height	2	3	
Traffic accident	3	2	
Other	5	4	0.794

#### Postoperative radiographic and functional outcomes

The follow-up rate was 100%, with a mean follow-up time of 24.4 months (range 12–27 months). Surgical parameters between the groups were similar in terms of time to surgery and estimated blood loss (*p* > 0.05) (Table 3). Group C was superior to group D in terms of procedure time (*p* < 0.05). All patients’ fractures healed at 6 months post-surgery, and the mean time to hardware removal was 20.4 weeks. Postoperative radiographic reduction grading showed that group D had higher “satisfactory” Tornetta and Matta ratings (all ratings of “excellent” and “good,” divided by total number of patients) than group C (77.78% vs. 60%, *p* = 0.046) [19]. Moreover, group D had no “poor” reduction ratings of the anterior ring (*p* = 0.019).

**Table 3:** Comparison of clinical outcomes

	Group C (*n* = 10)	Group D (*n* = 9)	*p*
Time to surgery (days)	3(2, 7)	4(3, 8)	0.107
Procedure time (min)	70.3 ± 11.8	98.7 ± 17.3	0.001
Blood loss (ml)	197.1 ± 15.6	209.2 ± 24.1	0.13
Tornetta and Matta grading			0.019
Excellent	1	1	
Good	3	6	
Fair	5	2	
Poor	1	0	
Satisfactory rate	4/10 (40%)	7/9, (77.78%)	0.095
Majeed score	79.25	91.33	0.029
Follow-up time (month)	20.5 (12–24)	23.2 (12–27)	

Group D received a higher Majeed rating, which was statistically significant at the 6-month follow-up visit (*p* = 0.029) (Table 3). However, no statistically significant difference was found at the 12-month follow-up visit (Table 3).

#### Complications

In group C, one patient developed non-union of the pubic ramus fracture, while all other patients achieved bony union at 3 months after surgery. Three patients (30%) in group C had postural pelvic tilt and sacroiliac joint instability at their 3-month post-surgery follow-up visit (Fig. 2). All three patients achieved bone healing after 3 months of strict bed rest. Two patients in each group experienced lateral femoral cutaneous nerve paralysis with symptom resolution achieved within 1 month after hardware removal. Two patients in each group developed asymptomatic heterotopic ossification. One patient developed a superficial infection due to the poor condition of soft tissue at the surgical site but achieved wound healing after debridement and dressing changes.

## Discussion

The aim of surgical treatment of unstable pelvic fractures is to correct the deformity, restore the anatomical structure of the pelvic ring, and promote early functional exercise. Previous studies have shown that the anterior and posterior rings account for 40% and 60% of the stability of the pelvis, respectively [20]. Therefore, simultaneous fixation of the anterior and posterior pelvic rings is often necessary to treat unstable pelvic fractures.

### Treatment of posterior pelvic ring fractures

Surgical fixation options for managing traumatic disruptions to the posterior pelvic ring included percutaneous sacroiliac screw fixation, plate fixation, and lumbosacral iliac screws. However, the most popular operative treatment option at present is percutaneous sacroiliac screw fixation, as it is associated with less trauma, less blood loss, and earlier mobilization than other treatments [21]. The fixation of double screws into the sacrum is associated with an increased risk of nerve injury due to significant variation in sacral anatomy [22].

Sacroiliac joint screw placement in the S1 vertebral body is the standard technique for posterior ring fixation [19, 23, 24]. Mears et al. showed that a single sacroiliac screw fixation can restore biomechanical stability similar to that of the complete pelvis in cadaveric models, under 10–350 N of vertical compressive load [25]. However, Yinger suggested that in unstable pelvic fractures, the placement of two sacroiliac joint screws increases stability against rotation and vertical displacement when the pelvic ring is loaded to 1000 N [26]. It is likely that the study by Mears et al. did not detect a difference between the techniques due to the use of inadequate vertical compressive load. In our study, we found no difference in anterior and posterior pelvic ring stability between the single- and double-screw fixation techniques when axial loading was less than 200 N; there are no differences between the single- and double-screw fixation in posterior stability under loads of 400 N and 800 N. However, for anterior pelvic ring stability, with loads of 400 N and 800 N, significant differences emerged between the two techniques, suggesting the superiority of the double-screw fixation technique.

### Treatment of anterior pelvic ring fractures

Traditionally, open reduction and plate fixation is the optimal treatment for anterior pelvic ring fractures, providing excellent stability and early mobilization. However, disadvantages include long operation times, large wound sizes and periosteal stripping areas, and increased bleeding. In addition, the risk of infection and re-operation exists, especially in obese patients or patients with a history of abdominal surgery. Furthermore, another technique employed in the treatment of these fractures is channel screw fixation, but it requires extensive training and a high level of surgical skill.

To address these treatment challenges, in 2009, Kuttert et al. performed the first anterior ring fixation for unstable pelvic fractures using INFIX [27]. INFIX has since become a popular treatment technique for unstable pelvic fractures, with reported benefits including reduced soft tissue injury, less blood loss, and low incidence of intraoperative iatrogenic nerve injury [7–9]. Studies investigating the biomechanics, anatomy, and clinical outcomes of INFIX have found that INFIX may provide adequate pelvic stability and achieve good clinical outcomes, despite its associated complications [27–30].

There are no data on the functional outcome of INFIX plus single versus INFIX plus double sacroiliac screw fixation technique for unstable pelvic ring injury (AO/OTA type C). We found that INFIX plus double sacroiliac screw fixation offered significantly better stability in the anterior pelvis than INFIX plus single sacroiliac screw fixation, under 400–800 N of axial loading. This result further supports the conclusion that the addition of a second screw improved the stability of the pelvis, which is consistent with the results of previous studies [18, 26, 31].

In addition, to investigate whether the INFIX plus double sacroiliac screw fixation technique improved functional outcomes clinically, 19 patients with unstable pelvic ring injury (AO/OTA type C) were retrospectively analyzed and followed up. We found that all fractures healed by the 6-month follow-up visit, with one patient who received INFIX plus single sacroiliac screw fixation experiencing non-union of the pubic ramus fracture.

In terms of joint stability, we did not find greater joint instability with single-screw fixation in our biomechanics study, but we did observe greater joint instability in the retrospective clinical analysis. In terms of nerve injury, two patients developed anterolateral numbness in the affected thigh, which is resolved by 3 months post-implant removal. Fang et al. reported an LFCN paralysis rate of 48.3% [32], which was much higher than the rate in our study. The reason for our finding may be improved surgical execution of the INFIX technique in the last decade and our use of pedicle screws with a smaller diameter (6.5 mm), compared with those used in Fang et al.’s study (7.3–10 mm). The limitations of this study are a retrospective study with a small sample size. A multicenter prospective studies with large sample size should be conducted in future study.

## Conclusion

INFIX plus double sacroiliac screw fixation showed greater stability in biomechanical analysis and better functional clinical outcomes in the treatment of unstable pelvic ring injury (AO/OTA type C) than INFIX plus single sacroiliac screw fixation.

## Data Availability

All data generated or analyzed during this study are included in this published article.
